# The Silence Around Myasthenia Gravis and Mood Disorders: An Editorial Reflection

**DOI:** 10.2174/0117450179486073260713093342

**Published:** 2026-07-15

**Authors:** Mauro G. Carta, Antonio E. Nardi

**Affiliations:** 1 Department of Medical Sciences and Public Health, University of Cagliari, 09124 Cagliari, Italy; 2 LABPR - Panic and Respiration Laboratory (LABPR), Institute of Psychiatry (IPUB), Federal University of Rio de Janeiro (UFRJ), 21941-908 Rio de Janeiro, Brazil

This article is intended as an editorial reflection grounded in clinical experience and consultation–liaison psychiatry practice rather than as a systematic review.

In consultation–liaison psychiatry, requests for psychiatric consultation for people affected by myasthenia gravis are relatively uncommon. Even more striking, however, is the limited body of evidence on the comorbidity between myasthenia gravis and mood or anxiety disorders.

The PubMed search reported below was conceived as a heuristic and illustrative exploration rather than as a systematic review strategy. Its purpose was not to provide a comprehensive synthesis of the literature, but rather to highlight the striking scarcity of studies explicitly addressing the relationship between myasthenia gravis and mood disorders.

A simple search on PubMed, the largest repository of biomedical literature, using the terms “Myasthenia Gravis and Major Depressive Disorder” (December 2025) yielded no results. In other words, no scientific articles were retrieved. For the sake of comparison, a parallel search for another disease of comparable prevalence, “Wilson’s Disease and Major Depressive Disorder”, returned 68 scientific publications. Admittedly, broader searches employing collateral strategies and extended sets of keywords do uncover a small number of papers [1], but the stark result of the initial query remains emblematic.

This absence naturally raises questions. Myasthenia gravis is a condition that profoundly compromises quality of life, following a chronic course marked by severe fatigue [2, 3], and often requires treatment with high-dose corticosteroids. These agents are well known to induce irritability and hypomanic symptoms [4, 5], while their tapering or discontinuation may precipitate severe depressive episodes [6, 7]. In myasthenia gravis specifically, high-dose steroid therapy has also been associated with anxiety and depressive symptoms [8].

It is also worth noting that an increased risk of depression has been discussed, although not definitively established, in association with immunosuppressive agents such as azathioprine, commonly included in therapeutic protocols [9, 10]. Beyond documenting prevalence, the literature should also address crucial clinical issues such as: which psychotropic medications are most appropriate for treating mood disorders in patients with myasthenia gravis, under what circumstances, and with what specific risks.

One critical point deserves emphasis. Steroids frequently induce dysphoric symptoms or mixed affective states. Importantly, the presence of depressive symptoms at high steroid dosages does not necessarily correspond to major depressive disorder in the strict nosographic sense. In such contexts, the risk of antidepressant-induced manic switching may be amplified, particularly given the limitations on the use of certain mood stabilizers in these patients.

From a broader consultation–liaison psychiatry perspective, the psychiatric burden associated with myasthenia gravis is likely multifactorial, involving neurobiological, immunological, pharmacological, and psychosocial mechanisms. Chronic inflammation, long-term corticosteroid exposure, functional impairment, uncertainty related to disease fluctuations, and social role disruption may all contribute to mood vulnerability.

Finally, converging evidence indicates that both autoimmune activation, an intrinsic feature of myasthenia gravis, and immune responses to infections, which are frequent in this condition, are associated with an increased risk of depressive episodes [11–13].

Why, then, does this silence persist?

Our own clinical encounter with myasthenia gravis may offer some interpretive clues.

Notably, we did not learn of this condition through formal consultation requests. Indeed, we have not received a single such request in more than forty years of clinical practice. Instead, we encountered three cases of myasthenia gravis referred to us without a diagnosis and labeled as “psychiatric cases difficult to treat”: two presumed cases of anorexia nervosa and one of presumed hysteria.

These individuals, affected by a profoundly disabling condition that was unrecognized not only by psychiatrists but by physicians more broadly, understandably did not develop a trusting or positive view of psychiatry. One of them contacted us again about a decade later during a clear depressive episode, immediately specifying that this time it was not “one of those absurd disorders that had been attributed years before.” This remark alone speaks volumes about the legacy of misdiagnosis and its impact on the therapeutic alliance.

Early diagnosis is essential for effectively addressing myasthenia gravis, especially in regions where preliminary epidemiological data suggest prevalence rates significantly higher than international averages [14]. The involvement and education of general practitioners are indispensable [15], as is the training of other specialists who often become the recipients of inevitable inappropriate referrals when the condition goes unrecognized.

For patients, this diagnostic odyssey is often long and burdensome. Its final stop is, all too often, the psychiatrist, who, lacking adequate recognition of the underlying disease, may end up assigning an inappropriate label to difficulties with swallowing, to early and often transient ocular symptoms, or to relentless fatigue. That inappropriate label, in turn, carries a heavy burden of stigma.

For this reason, a central message for individuals affected by myasthenia gravis must be that there is no shame in seeking help when anxiety or depressive symptoms intensify. There is a pressing need to develop networks of psychotherapeutic support comprising clinicians capable of guiding patients appropriately when pharmacological treatment appears indicated. Consultation–liaison psychiatry has grown substantially, and consultation units now exist in major general hospitals. These should become the natural points of contact for psychological or psychiatric support.

## LIMITATIONS

This contribution has several limitations that should be explicitly acknowledged. First, it does not aim to present a systematic review of the literature, but rather to offer an editorial reflection informed by clinical experience in consultation–liaison psychiatry. The PubMed observations presented here are heuristic in nature and intended to illustrate the relative invisibility of the topic within mainstream biomedical indexing rather than to provide quantitative evidence.

Second, many of the considerations proposed remain exploratory and should therefore be interpreted cautiously. Paradoxically, however, the awareness of these methodological limitations further underscores the fragility, fragmentation, and relative absence of the current literature on this topic.

Finally, the reflections presented here should be understood as an invitation to develop more rigorous epidemiological, neurobiological, and consultation–liaison psychiatry research on the relationship between myasthenia gravis and mood disorders.

Breaking the silence around myasthenia gravis and mental health is not merely an academic task; it is also an ethical imperative.
